# Non-parametric MRI Brain Atlas for the Polish Population

**DOI:** 10.3389/fninf.2021.684759

**Published:** 2021-10-06

**Authors:** Damian Borys, Marek Kijonka, Krzysztof Psiuk-Maksymowicz, Kamil Gorczewski, Lukasz Zarudzki, Maria Sokol, Andrzej Swierniak

**Affiliations:** ^1^Faculty of Automatic Control, Electronics and Computer Science, Department of Systems Biology and Engineering, Silesian University of Technology, Gliwice, Poland; ^2^Biotechnology Center, Silesian University of Technology, Gliwice, Poland; ^3^Department of Medical Physics, Maria Sklodowska-Curie National Research Institute of Oncology Gliwice Branch, Gliwice, Poland; ^4^Department of Radiology, Maria Sklodowska-Curie National Research Institute of Oncology Gliwice Branch, Gliwice, Poland

**Keywords:** sub-population brain template, non-parametric atlases, statistical parametric mapping, MRI, tissue probability maps

## Abstract

**Introduction:** The application of magnetic resonance imaging (MRI) to acquire detailed descriptions of the brain morphology *in vivo* is a driving force in brain mapping research. Most atlases are based on parametric statistics, however, the empirical results indicate that the population brain tissue distributions do not exhibit exactly a Gaussian shape. Our aim was to verify the population voxel-wise distribution of three main tissue classes: gray matter (GM), white matter (WM), and cerebrospinal fluid (CSF), and to construct the brain templates for the Polish (Upper Silesian) healthy population with the associated non-parametric tissue probability maps (TPMs) taking into account the sex and age influence.

**Material and Methods:** The voxel-wise distributions of these tissues were analyzed using the Shapiro-Wilk test. The non-parametric atlases were generated from 96 brains of the ethnically homogeneous, neurologically healthy, and radiologically verified group examined in a 3-Tesla MRI system. The standard parametric tissue proportion maps were also calculated for the sake of comparison. The maps were compared using the Wilcoxon signed-rank test and Kolmogorov-Smirnov test. The volumetric results segmented with the parametric and non-parametric templates were also analyzed.

**Results:** The results confirmed that in each brain structure (regardless of the studied sub-population) the data distribution is skewed and apparently not Gaussian. The determined non-parametric and parametric templates were statistically compared, and significant differences were found between the maps obtained using both measures (the maps of GM, WM, and CSF). The impacts of applying the parametric and non-parametric TPMs on the segmentation process were also compared. The GM volumes are significantly greater when using the non-parametric atlas in the segmentation procedure, while the CSF volumes are smaller.

**Discussion and Conclusion:** To determine the population atlases the parametric measures are uncritically and widely used. However, our findings suggest that the mean and parametric measures of such skewed distribution may not be the most appropriate summary statistic to find the best spatial representations of the structures in a standard space. The non-parametric methodology is more relevant and universal than the parametric approach in constructing the MRI brain atlases.

## 1. Introduction

The application of magnetic resonance imaging (MRI) to acquire detailed descriptions of the brain morphology *in vivo* is a driving force in brain mapping research. The terms *brain atlas* and *brain template* are used interchangeably in the corresponding literature to date, and while they may have different meanings in some situations, many papers do not make this clear. Additionally, the term: *registration target* as the element representing a standard space in the image specified modalities obtained for some population can be used (Dickie et al., 2017). Atlas may also contain the datasets with the tissue proportion maps. These maps show the spatial distribution of tissue probability in the standard space. Brain atlases are constructed with the use of parametric or non-parametric statistics. Parametric statistics are based on a probability theory that relies on many assumptions, such as a parametric distribution at each spatial location. Thus, the parametric approach provides a set of parameters (simple and easy to interpret); for example, the mean plus/minus double standard deviation is used to estimate the 95 percent limit of each voxel value (Dickie et al., 2015). The non-parametric approach avoids making hard to verify assumptions, requires only minimal assumptions for validity, deals with the multiple comparisons problem, and can be applied when the assumption of normality is untenable. Non-parametric permutation tests are exact, distribution-free, and adaptive to underlying correlation patterns in the data (Pantazis et al., 2004).

Widely adopted methodology used in the MRI atlas creation process relies on the Gaussian assumptions for representing the underlying population distributions of cerebrospinal fluid (CSF), gray matter (GM), and white matter (WM)—thus, most available atlases are based on the parametric statistics (mean and standard deviation), being designed to provide a standard space for voxel-wise analyses or to support tissue/ROI volume segmentation (Dickie et al., 2017). However, the empirical results indicate that the brain tissue content distributions do not exhibit exactly a Gaussian shape (Dickie et al., 2015). The non-parametric data distribution is much more common in the field of MRI data analysis (Despotović et al., 2015; Dickie et al., 2015, 2017; Kim et al., 2016; Dadar and Collins, 2021), but there are, in fact, its two aspects:

- The non-parametric pixel intensity distribution in different MR images—it relates to the classification process in the image segmentation algorithms (modeling the class intensities based on either parametric or non-parametric finite mixtures) (Avants et al., 2011; Dadar and Collins, 2021). This issue is essentially concerned with the intensity distribution of a single subject (image).
- The non-parametric population distributions of the structure content data (the population information on the tissue content) in the standardized space—these issues relate to the template creation process (usually by simply averaging the segmented tissue maps from the population to obtain the representations of the structures in a standard space) (Dickie et al., 2015, 2017).

In both cases, the applicability of the non-parametric methodologies is being investigated (Cocosco et al., 2003; Lee et al., 2009; Dickie et al., 2017). The Gaussian mixtures models seem to fail to represent the MRI data in the segmentation process (Lee et al., 2009); therefore, other approaches have been developed. For instance, Cocosco et al. (2003) have presented a non-parametric method to segment the brain images contaminated by the partial volume effects, whereas Ashburner and Friston (2005) have assigned multiple Gaussians for each pure tissue class to fit the non-Gaussian intensity distribution of the pure tissue class.

However, the bias introduced by the population distributions of the tissue content maps in the process of determining the atlases is still poorly covered and explained (Dickie et al., 2017), though expected (LeWinn et al., 2017). Dickie et al. (2015, 2017) have presented the first non-parametric brain MRI atlas and have shown clearly, that the voxel-wise proportions of GM in older subjects - healthy and with the neurodegenerative diseases, do not follow a Gaussian distribution and that the statistical method used for the construction of brain MRI atlases should be selected taking into account the population.

The human brain is highly variable among the phenotypically different groups (i.e., race) with fundamental genetic and environmental disparities in brain morphology and micro-structure (e.g., shape, size, and volume) (Takahashi et al., 2011; Liang et al., 2015; Skorupa et al., 2017; Kijonka et al., 2020). Thus, the brain atlases created for a specific population cannot be used in other populations, because the genotypic and phenotypic differences may cause inaccurate measurements, comparisons, and interpretations of the results (Tang et al., 2010). Because the Polish population is one of the most ethnically homogeneous in Europe (its haplogroup composition, though similar to other European populations, has, however, some statistically significant differences reflecting the genetic regional specificities and the migration history, Grzybowski et al., 2007; Grochowalski et al., 2020), the disturbances from the ethnic factor can be avoided.

The aim of this study was to verify the population voxel-wise distribution of three main morphological classes (GM, WM, and CSF) and to construct the brain templates for the Polish (Upper Silesian) population with the associated non-parametric tissue probability maps (TPMs) taking into account the sex and age influence. The non-parametric atlases were generated from 96 brains of the ethnically homogeneous, neurologically healthy, and radiologically verified group examined in a 3-Tesla MRI system. The validity of using the parametric statistics in the mapping of the high-resolution stereotaxic tissue proportions maps in the atlas determination process has been discussed.

## 2. Materials

### 2.1. Human Subjects

The study sample was drawn from a database of 100 volunteers selected as the control group (homogeneous in terms of its ethnicity—Caucasian cohort) from the Upper Silesia region in Poland. All participants underwent full brain MRI examinations in the Radiology and Diagnostic Imaging Department of Maria Sklodowska-Curie National Research Institute of Oncology, Gliwice branch, between 2013 and 2014. The MR images were validated for the lack of pathology by an experienced radiologist. The inclusion criteria to the studied sub-population involved the age above 18 and a good health status, i.e., the absence of acute or chronic diseases (no neurological disorders or surgical history) and no pathologies in the central nervous system in the MR images. Four subjects were excluded from the investigation due to the presence of the silent gross brain lesions (three cases) and due to the image artifacts (1 case), resulting in a final sample of 96 subjects, aged from 20 to 66 years (median age 37.0 years, 25*th* percentile 29.0 years, 75*th* percentile 50.0 years). Although the subjects were randomly chosen among the Polish population, the age and sex distributions were found to reflect the characteristics of the whole population (groups description is presented in Table 1), as revealed from the Statistical Atlas of Slaskie Voivodeship edited by Central Statistical Office of Poland (http://stat.gov.pl/en/topics/other-studies/cities-voivodship/statistical-atlas-of-slaskie-voivodship). The studied group was divided into the following sub-groups—two of both sexes (nM = 42 males and nF = 54 females) and two age-related: the younger sub-group, aged 20–35 (nY = 46 subjects) and the older one, aged 36–66 (nO = 50). According to these divisions, the appropriate templates were obtained—for the studied group and for the sub-groups—and presented in Table 1.

**Table 1:** Subpopulation templates names, description, and cardinality.

**Sub-atlas description**	**Cardinality**	**Median**	**Age percentiles**	**Range**
	**[volunteers]**	**[Age]**	**(25*th; 75*th)**	**[Age]**
All volunteers (All)	96	37.0	29.0; 50.0	[20–66]
Females from all volunteers (Women)	54	38.5	29.3; 50.0	[24–66]
Males from all volunteers (Men)	42	36.0	29.0; 43.0	[20–58]
Old (>35 years old) group (Old)	50	49.0	40.0; 53.5	[36–66]
Young (< =35 years old) group (Young)	46	29.0	25.0; 32.0	[20–35]

The validation T1-weighted data (3D spoiled gradient-echo sequence) was also collected to be suitable for the objective and unbiased comparison of the segmentation process. The validation group included 75 volunteers with the range of 18–65 years (median age 27 years; 25*th* percentile 23.0 years, 75*th* percentile : 38.5 years); the female to male ratio was 53:22. The ethnicity of the validation group and the scanner used for the study were consistent with the main data. The obtained examinations were reviewed for exclusion of pathology in the central nervous system in the MR images.

### 2.2. Data Acquisition

A Philips 3T Achieva MRI scanner (Radiology and Diagnostic Imaging Department, Maria Sklodowska-Curie National Research Institute of Oncology, Gliwice branch Poland) with a dedicated, 8 channel head coil was used for data collection. A 3D spoiled gradient-echo sequence was applied (T1-FFE) with TR/TE/flip angle of 20 ms/2.9 ms/20 deg and the SENSE technique of parallel imaging with a sensitivity encoding. The acquisition matrix was 256 × 256 in the *x* and *y* dimensions yielding a voxel of 1 × 1 mm. The images were acquired using spacing between the slices of 1 mm, and at a slice thickness of 2 mm. To obtain a T2-weighted scan, a 2D turbo spin-echo (TSE) SENSE sequence with TE = 80 ms was employed. TR varied, depending on the number of slices, but always it was longer than 2,500 ms. The acquisition matrix was adjusted to the brain size; however, the voxel size of 1 × 1 × 1 mm was always maintained.

An experienced certified radiologist evaluated all MR images to identify the image artifacts and exclude the presence of morphological pathologies (silent gross brain lesions). The original MR images encoded in DICOM were converted to the NIfTI format supported by all major software packages, like FSL (Jenkinson et al., 2012), SPM (Friston et al., 2007), ANTs (Avants et al., 2008, 2011, 2014), and many other brain imaging tools.

## 3. Methods

**Figure 1:**
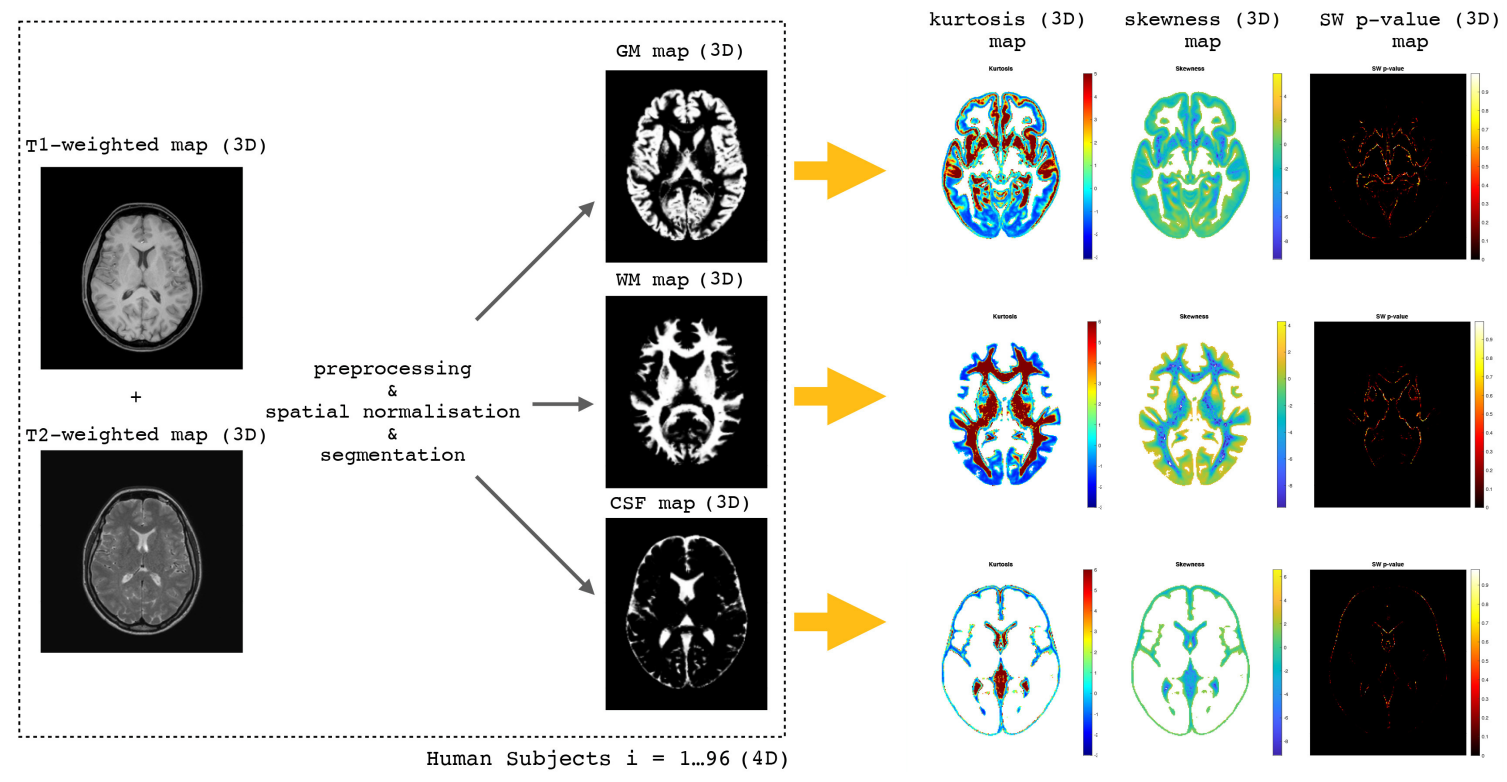
The flow chart of the whole methodology starting from T1-weighted and T2-weighted maps (3D) through the preprocessing, spatial normalization, and segmentation steps to obtain WM, GM, and CSF 3D maps. Having 96 human subjects we obtain 4D maps which are used to perform voxel-wise distributions of skewness, kurtosis, and SW test *p*-values.

A short description of the methodology adopted in the present study is shown in Figure 1.

### 3.1. Image Pre-processing

#### Image Denoising

To increase the quality and performance of the applied methods, the MR images were denoised using the Smallest Univalue Segment Assimilating Nucleus (SUSAN) 3D noise reduction tool (Smith and Brady, 1997) available in FSL (Jenkinson et al., 2012), thus using the same method as in our previous study (Kijonka et al., 2020). The DICOM data files were processed in a full 3D mode taking into account the brightness threshold differences separately for each image and each volunteer. The image filtering procedure was performed by the SNR (signal-to-noise ratio) and CNR (contrast-to-noise ratio) values determination. The brightness threshold was optimized to be higher than the noise level and less than the contrast of the underlying image. The Gaussian mask was set to a default size of 3 × 3 × 3.

#### Nonuniform Intensity Correction

The N3 method was used to correct intensity non-uniformity in both T1- and T2-weighted images. The non-parametric non-uniform intensity normalization (N3) (Sled et al., 1998) with the parameters (the maximum number of iterations equal 100, the stopping of 0.0001 and the kernel full-width, half maximum (FWHM) of 0.1 to sharpen the histogram) using the *nu_correct* tool from the MINC package was applied (Collins et al., 1994). This part of the data pre-processing was done according to the methodology of Fonov et al. (2011).

#### Intensity Normalization

To obtain the same distribution and the range of the intensity histogram of the MR images as for the MNI-ICBM 152 template (Fonov et al., 2011), a linear normalization of the intensity (a single linear histogram scaling) was applied for each DICOM data set. Such intensity normalization is based on the method proposed by Nyúl and Udupa (1999). It offers a simple way of transforming the images non-linearly so that there is a significant gain in the similarity of the resulting images. In a training stage, executed only once for a given protocol and a body region, the parameters of the standard scale are determined. Thus, certain landmarks of a standard histogram (for the head region and the protocol under consideration) are estimated from a given set of volume images. This procedure is followed by a transformation stage (for each given volume image) to map the candidate volumes' histograms onto the standard histogram scale. In other words, it means that the actual intensity transformation from the intensity scale of the input volume image to the standard scale is computed by mapping the landmarks determined from the histogram of a given volume image to those of the standard histogram.

### 3.2. Segmentation of the Tissue Proportion Maps

The unified segmentation procedure was applied in Statistical Parameter Mapping (SPM) software (Wellcome Department, University College, London, UK) (Ashburner and Friston, 2005). The two-channel (T1- and T2-weighted) segmentation mode was used and the classification parameters were optimized in a similar manner as in our previous work (Kijonka et al., 2020). The SPM steps were adopted in the present study, and the GM, WM, and CSF proportion maps were determined for all subjects in the common brain MRI processing pipeline.

### 3.3. Registration Targets (Standard Spaces)

The subjects selected as normal (in terms of the inclusion criteria) were used to create the T1-weighted registration targets [Polish (Upper Silesian) standard spaces] according to the methodology by Dickie et al. (2015). Five registration standard spaces were obtained: one from all subjects, two from the sex sub-groups, and two from the age sub-groups (see section 2.1). Moreover, the standard spaces for the T2-weighted were also generated.

To spatially normalize all subjects to our standard T1-weighted registration target, we devised a “non-linear surface” spatial normalization as described by Dickie et al. (2015) as “Nsurf” using ANTs (version 2.3.2) SyN diffeomorphic registration method (Avants et al., 2008, 2011). The critically important aspect in the voxel-wise tissue distribution analysis is to preserve the variance of the data by performing the appropriate spatial normalization. A linear spatial normalization maintains within the brain variance, e.g., the ventricle size, between the subjects but does not always adequately account for the head size. On the other hand, the nonlinear spatial normalization generally accounts for the head size differences but removes the brain variance between the subjects. “Nsurf” spatial normalization fully accounts for the head size differences while also maintains the within brain variance of interest, for example, the size of lateral ventricle and cortical thickness (Dickie et al., 2015). The transformation matrices obtained in the appropriate normalization procedure (to the T1-weighted registration target) were applied to the T2 modality to spatial positioning within a single subject.

### 3.4. Voxel-Wise Distributions of the WM, GM, and CSF Data

After the spatial normalization of the WM, GM, and CSF proportion maps for all sub-groups, the data distributions of the three-dimensional tissue maps were tested using the Shapiro-Wilk test (Ghasemi and Zahediasl, 2012; Dickie et al., 2015). This test determines whether or not the data derived from the analyzed population reveal a Gaussian distribution, and the parametric measures are appropriate to describe it. The results of the Shapiro-Wilk test were presented as a three-dimensional matrix showing the results at each voxel in a standard space for each structure (GM, WM, and CSF). Moreover, we calculated the maps of kurtosis and skewness to check the central and outer appearance of the data distribution and the data symmetry in the WM, GM, and CSF voxels (Dickie et al., 2015). The kurtosis was calculated using the Fisher's definition (Dorić et al., 2007):


(1) $$K\;=\frac{\frac{1}{n}\sum_{i=1}^{n}{\left(x_{i}-\mu \right)}^{4}}{\sigma^{4}}-3$$

where *x*_*i*_ are the values of the variable x, μ is the mean of the variable x, *n* is the number of data points, and σ is the standard deviation of the variable x. Then, the value 3.0 is subtracted from the result to give 0.0 in case of the normal distribution. The skewness is calculated using the equation:


(2) $$S\;=\frac{\frac{1}{n}\sum_{i=1}^{n}{\left(x_{i}-\mu \right)}^{3}}{\sigma^{3}}$$

where μ is the mean, and σ is the standard deviation, and *n* is the number of data points. In computing the skewness, σ is computed with *n* in the denominator rather than (*n*−1).

### 3.5. The WM, GM, and CSF Proportions for the Parametric and Non-parametric Atlases

**Figure 2:**
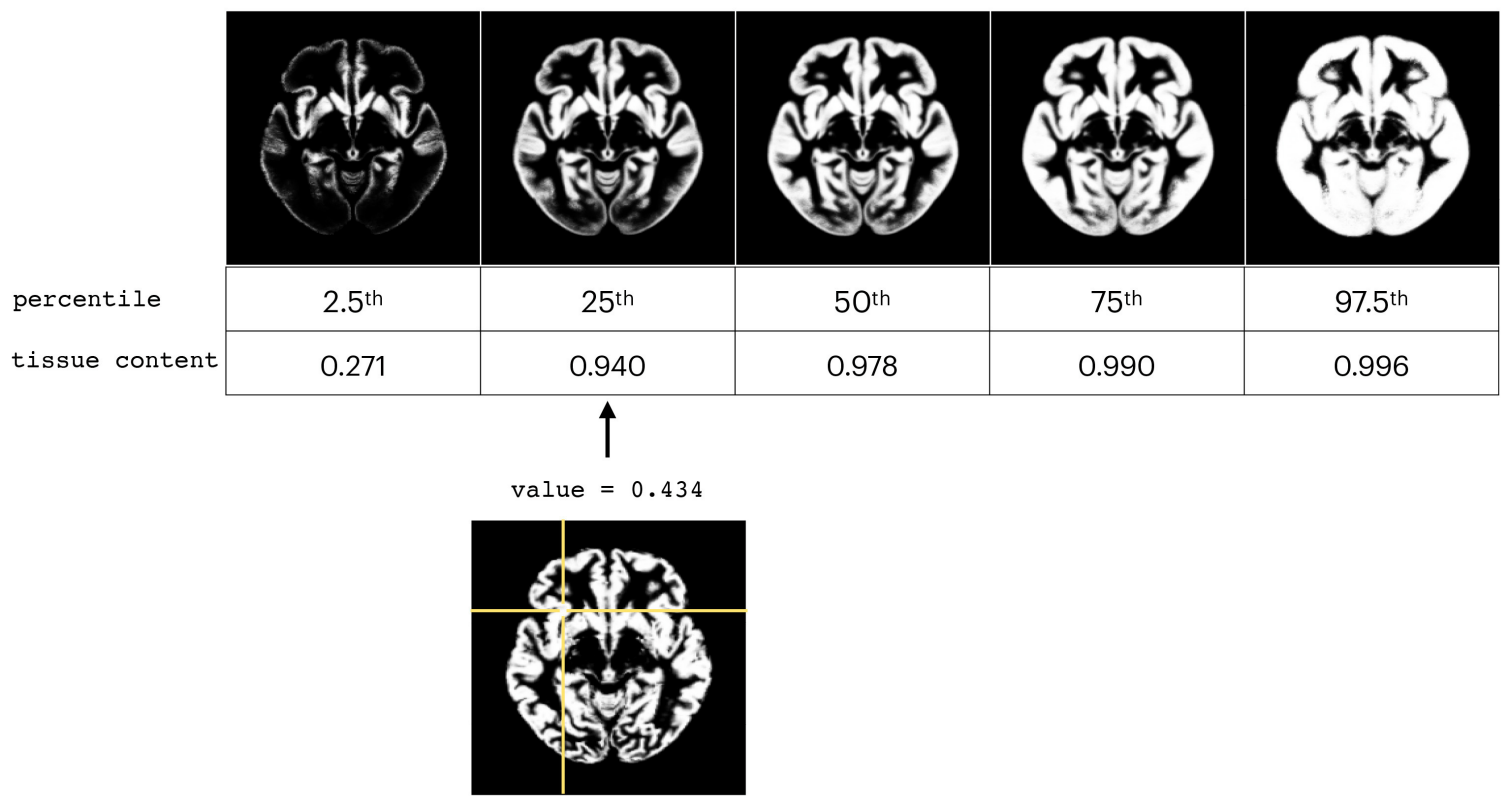
Presentation of different levels of percentile maps for GM atlas **(top)** and comparison with one individual subject **(bottom)**.

The WM, GM, and CSF proportion images from the analyzed sub-groups (described in section 2.1) selected as the normal subjects (in terms of the inclusion criteria) were used to create the order-based non-parametric atlases. The example of the different levels of percentile maps for GM atlas is presented in Figure 2.

The percentile ranks (*t*^*th*^ percentile) were calculated as follows (3) (Dickie et al., 2015):


(3) $$\begin{array}{l} (t/100)=j+g\\ \;y=\frac{1}{2}(x_{j}+x_{j+1})\;if\;g=0\\ \;y=x_{j+1}\;if\;g>0 \end{array}$$

where *n* is the number of subjects, *j* is the integer part of the left hand side of the first equation, *g* is the fractional part of the left hand side of the first equation, *y* is the *t*^*th*^ percentile, and *x*_1_, *x*_2_, ..., *x*_*n*_ are the ordered values of each brain volume. The atlases were generated for the three investigated classes: WM, GM, and CSF. Moreover, we calculated the atlases for all investigated subjects and for the sex and age sub-populations.

The non-parametric approaches more commonly rely on rank or order statistics being the percentiles of a distribution. Percentile indicates the percent of a distribution that is equal to or below it. Their calculation is not based on the arithmetic mean, which should not be used for the skewed data. They (and therefore also the percentile rank classes) offer an alternative to mean-based quotients. Generally speaking, percentiles are not as strongly influenced by extreme values of the distribution as the mean value. The *t*^*th*^ percentile is a value, such that at most *t%* of the ordered measurements are less than this value, and at most (100−*t*)% are greater (Hyndman and Fan, 1996). Thus, instead of a mean value, the median or the 50*th* percentile is used, whereas the difference between the upper quartile (75*th* percentile) and the lower quartile (25*th* percentile) is called the interquartile range and is the non-parametric alternative to the standard deviation for describing the spread.

We also calculated the parametric (mean) tissue proportion maps for the investigated sub-groups. The mean and median (non-parametric 50*th* percentile) tissue proportion maps were compared using the percentage differences in the structure content. The percentage difference comparisons were presented as matrices for each structure (GM, WM, and CSF) TPMs.

The TPMs volumes of GM, WM, and CSF were calculated by counting the probability in the voxels for the sex- and age-related TPMs.

To test the TPMs against the null hypothesis that the two images have the same content mean ranks we used the Wilcoxon signed-rank test (Demidenko, 2004; Rey and Neuhäuser, 2011; Thor et al., 2013). The Wilcoxon test is a paired difference non-parametric statistical test in which the null hypothesis assumes that the difference between the paired data (the TPMs voxels for each structure) follows a symmetric distribution around zero (Rey and Neuhäuser, 2011).

Moreover, to compare the TPMs under the null hypothesis that two tissue maps have the same distribution of the structure content we used the Kolmogorov-Smirnov test (Demidenko, 2004). This group test reduces to a comparison of only two groups of gray level distributions and it does not use the content or structural information in the images.

The *p* < 0.05, a predetermined significance level, were accepted as indicating that the observed result would be highly unlikely under the null hypothesis.

### 3.6. Comparison Between the Classification Results Using Parametric and Non-parametric Atlases

We also compared the volumetric features (the volumes of GM, WM, and CSF) calculated using the non-parametric and standard parametric templates in the SPM segmentation procedure. For each subject in the validation cohort, the volumes of GM, WM, and CSF were obtained by counting the probability in the voxels segmented using different templates. The volumetric parameters segmented using the parametric and non-parametric Polish atlases were compared using a paired-sample Wilcoxon's signed-rank test.

## 4. Results

### 4.1. Voxel-Wise Distribution of WM, GM, and CSF

**Figure 3:**
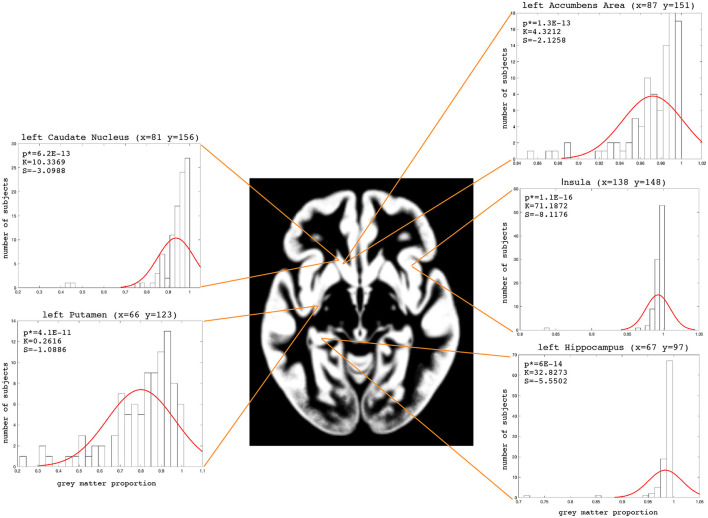
Axial slice (z = 72) for the median gray matter map. The histograms with the skewness (S), kurtosis (K), and Shapiro-Wilk test *p*-values (p*) for the left Caudate Nucleus, left Putamen, left Accumbens Area, left Hippocampus and Insula.

**Figure 4:**
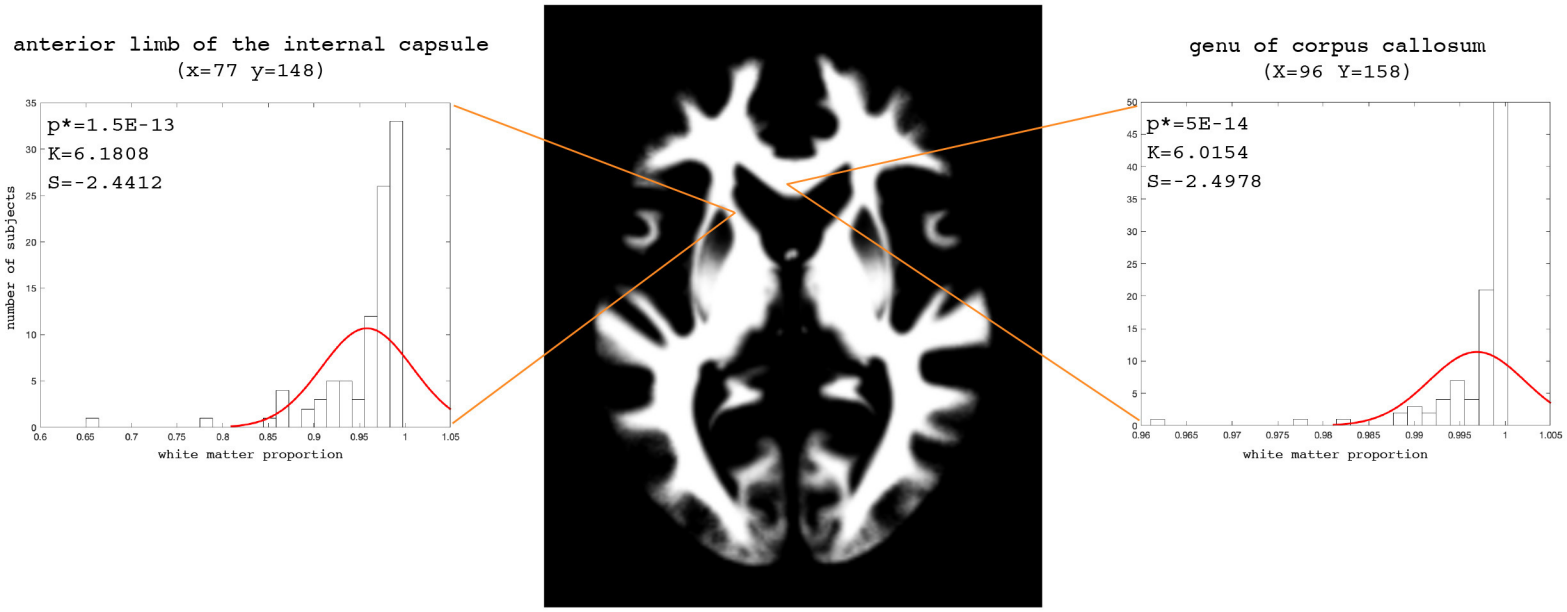
Axial slice (z = 83) for the median white matter map. The histograms with the skewness (S), kurtosis (K), and Shapiro-Wilk test *p*-values (p*) for the anterior limb of the internal capsule and genu of the corpus callosum.

**Figure 5:**
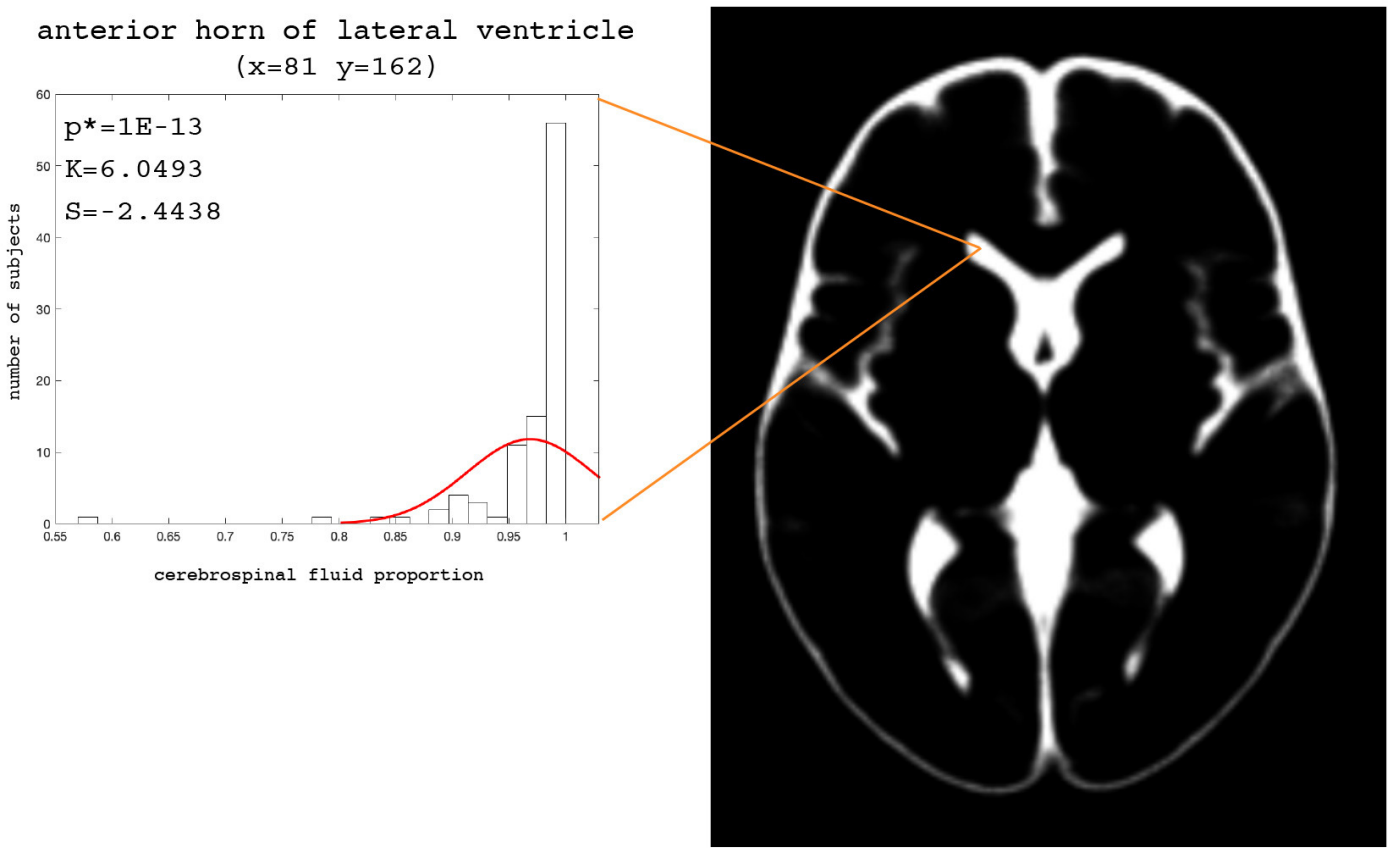
Axial slice (z = 82) for the median CSF map. The histograms with the skewness (S), kurtosis (K), and Shapiro-Wilk test *p*-values (p*) for the anterior horn of the lateral ventricle.

The examples of the axial slices for the median maps of the gray matter, the white matter, and CSF are shown in Figures 3–5. The randomly selected examples of the population distributions of the structure content in the regions of GM (Figure 3: left caudate nucleus, left putamen, left accumbens, insula, and left hipocampus), WM (Figure 4: anterior limb of the internal capsule and genu of corpus callosum) and CSF (Figure 5: anterior horn of lateral ventricle) are also shown. As seen, the example histograms of the structure proportions are composed of the asymmetrical left skewed (*Skewness* < 0) and leptokurtic distributions (*Kurtosis*>0) that are markedly non-Gaussian (Figures 3–5). The Gaussian estimations of the histograms are also added as the red curves (Figures 3–5) for the sake of comparison. The values of the kurtosis and the skewness for a normal distribution equal to 0. The Shapiro-Wilk test confirms the non-Gaussian distributions observed in the presented histograms (*p* < 0.05; Figures 3–5).

**Figure 6:**
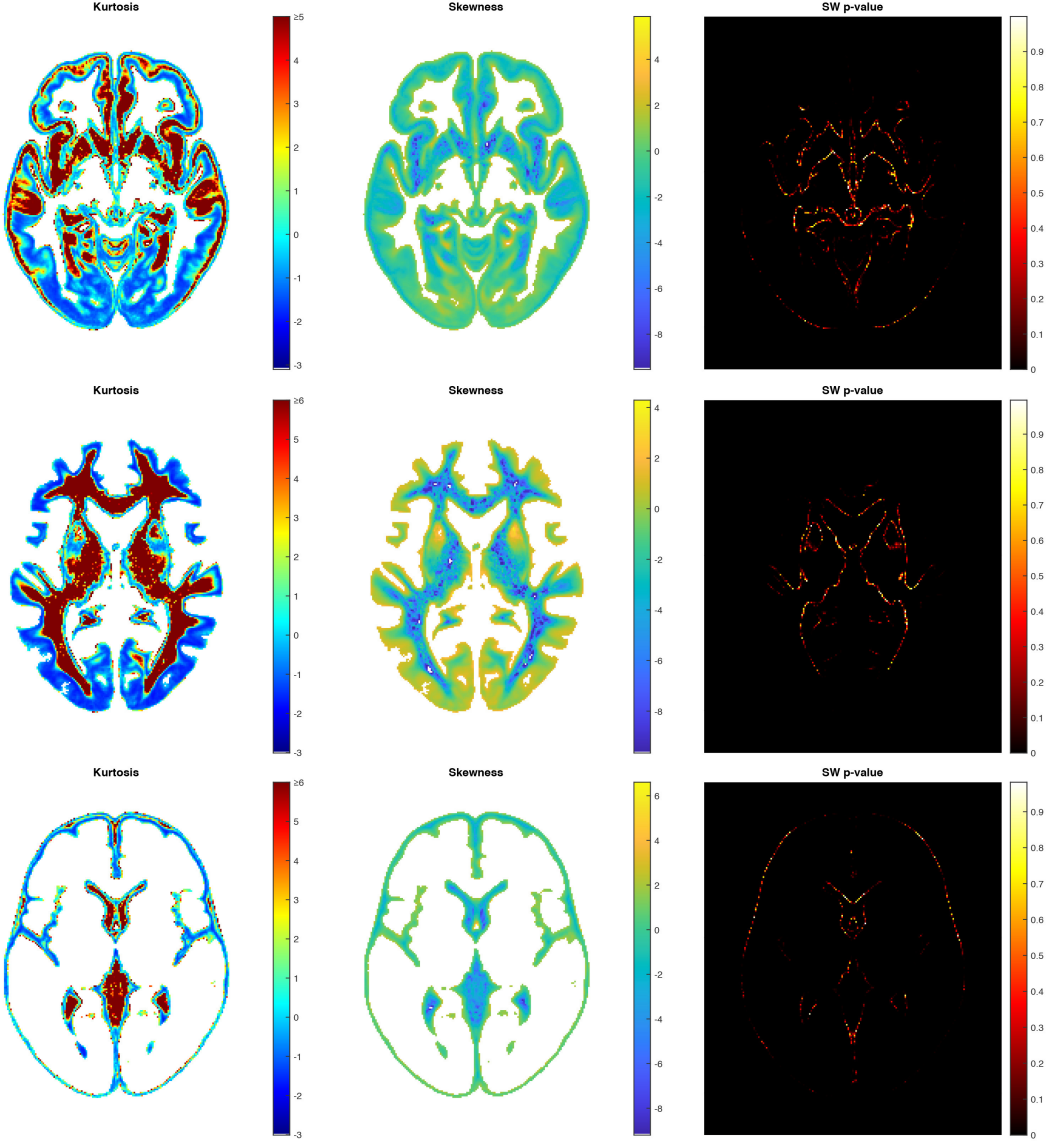
Axial slices of the kurtosis, the skewness and the Shapiro-Wilk test *p*-value map for GM **(top)**, WM **(middle)**, and CSF **(bottom)**.

To assess the WM, GM, and CSF distributions quantitatively throughout the brain, we calculated the voxel-based 3D maps of the kurtosis, skewness, and a Shapiro-Wilk test for the studied population. The previously presented coordinates for the axial slices have also been applied to these maps in each structure to show the example of spatial variability in the structure distributions (Figure 6). The obtained images (skewness, kurtosis, *p*-value, proportion maps) were masked using the threshold at 5% of the structure content to present the results only in those areas where the structures exist spatially significantly. The kurtosis and skewness maps peripherally reveal in the analyzed structure a right-skewed platokurtic distribution, whereas the left-skewed leptokurtic distribution is observed in the internal part of the structure. The voxels located between these areas are of a symmetrical distribution with the kurtosis values close to 0. Only the regions at the border of the tissue proportion maps show a normal population distribution with a Shapiro-Wilk test *p*>0.05 (Figure 6).

Moreover, Table 2 collects the middle values (medians) of skewness and kurtosis as well as the *p*-values (Shapiro-Wilk test) for the investigated tissue maps. These median values indicate that in each brain structure (regardless of the studied subpopulation), the data distribution is leptokurtic (Table 2). The distribution is right-skewed for GM and CSF, while in WM it is left-skewed. The *p*-values of the Shapiro-Wilk test for the distributions of the WM, GM, and CSF proportions indicate that the observed results would be highly unlikely under the null hypothesis and are generally non-Gaussian (Table 2).

**Table 2:** The medians of the Kurtosis, Skewness, and Shapiro-Wilk test *p*-values (p^*^) for GM, WM, and CSF (in columns) for the studied groups (All, Men, Women, Young, and Old).

	**Gray matter**			**White matter**			**Cerebrospinal fluid**		
**Group**	**Kurtosis**	**Skewness**	*p* ^*^	**Kurtosis**	**Skewness**	*p* ^*^	**Kurtosis**	**Skewness**	*p* ^*^
All	1.029	−0.091	3.0E-09	3.921	0.602	1.0E-11	7.894	2.652	6.4E-13
Men	0.748	−0.059	6.7E-13	2.842	0.582	5.4E-14	5.014	2.243	4.1E-15
Women	0.873	−0.106	8.0E-12	3.205	0.597	1.7E-13	6.095	2.404	1.9E-14
Young	0.890	−0.164	1.5E-12	2.907	0.616	8.2E-14	5.728	2.382	5.6E-15
Old	0.756	−0.012	4.3E-12	3.146	0.556	1.4E-13	5.350	2.262	1.6E-14

**Figure 7:**
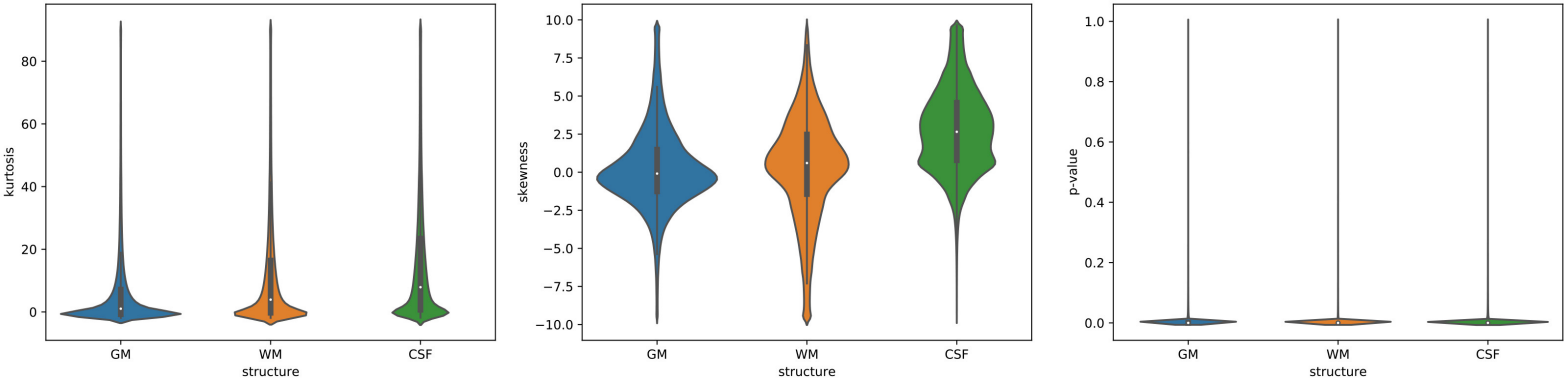
The violin plots of the data distribution (**left**: kurtosis, **middle**: skewness, **right**: Shapiro-Wilk test *p*-values) across the entire brain (GM, WM, and CSF).

To visualize the full distribution of the data (kurtosis, skewness, and *p*-values) across the entire brain, the violin plots were calculated (Figure 7). The violin plots estimate the data distribution by using a kernel density function (Weissgerber et al., 2017). The kurtosis values are stretched toward the values for the leptokurtotic distributions (Figure 7, left). The voxels skewness for GM is slightly shifted to the negative side, whereas for WM and CSF the positive shift is seen (Figure 7, middle). Finally, the *p*-values are clustered around 0 for all structures (Figure 7, right). Thus, these results are consistent with the data from Table 2.

### 4.2. Parametric and Non-parametric Atlases

**Figure 8:**
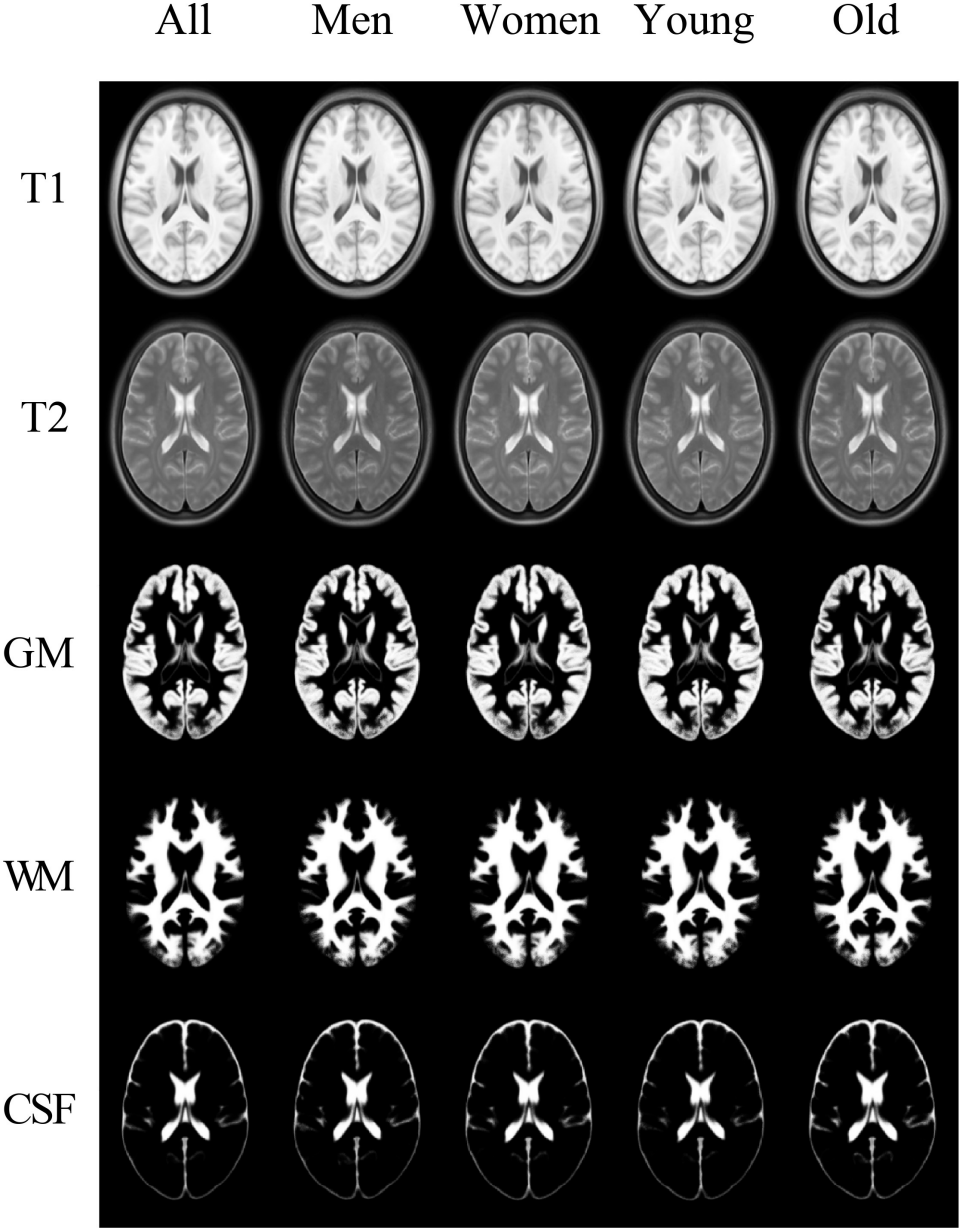
The templates in the columns: All, Men, Women, Young, and Old. In the rows: the registration targets of T1 and T2 and the templates for GM, WM, and CSF.

The examples of the axial slices for the non-parametric (order-based 50*th* percentile) atlases of the tissue proportions maps in the appropriate sex- and age-related sub-groups for the healthy Polish population are shown in Figure 8. The standard parametric (mean) tissue proportion maps for the investigated sub-groups were also calculated to compare the results obtained by the different measures in the atlas calculation process.

The TPMs volumes of GM, WM, and CSF for the sex- and age-related sub-populations were also calculated and Table 3 shows the results obtained for the parametric and non-parametric maps. The median and mean (tissue proportions) maps were compared using the Wilcoxon signed-rank test and the results of the comparisons are also in Table 3. The statistically significant differences were found between the maps obtained using the different statistical measures (*p* < 0.05, Table 3). For all subgroups, the GM volumes calculated from the tissue probability maps (TPMs) obtained with the non-parametric measure were higher than the values estimated with the parametric methods. On the other hand, the TPM volumes of WM and CSF gotten by the non-parametric methods were lower, regardless of the analyzed sub-population.

**Table 3:** The results of the Wilcoxon signed-rank test p-values (p*) of the parametric and non-parametric TPMs comparison.

	**Gray matter**			**White matter**			**Cerebrospinal fluid**		
	**Volume [ml]**			**Volume [ml]**			**Volume [ml]**		
**Group**	**Median**	**Mean**	*p* ^*^	**Median**	**Mean**	*p* ^*^	**Median**	**Mean**	*p* ^*^
All	968.21	946.22	1.4E-309	674.69	699.66	≈0	290.24	334.38	≈0
Men	954.01	933.75	2.6E-219	680.56	704.84	≈0	285.64	327.91	≈0
Women	978.21	956.42	≈0	671.70	695.54	≈0	297.31	340.36	≈0
Young	1008.8	983.5	≈0	665.92	691.11	≈0	270.91	313.68	≈0
Old	929.29	912.37	5.5E-71	685.36	707.99	≈0	312.07	354.02	≈0

*The TPM volumes of GM, WM and CSF obtained for the parametric (mean) and non-parametric (median) methods. The rows present the values for each group (All, Men, Women, Young and Old)*.

The Sex and Age subgroups' non-parametric TPMs were also compared using the Kolmogorov-Smirnov test to find the differences resulting from the specificity of the investigated subpopulation. The results (Tables 4, 5) show that the maps for the sub-populations differ significantly and the TMPs volumes differences are even stronger than the impact of the applied statistical measure (Table 3).

**Table 4:** The results of the Kolmogorov-Smirnov test *p*-values of the comparisons of the Sex subgroups' non-parametric TPMs.

	**Volume [ml]**		
	**Women TPMs**	**Men TPMs**	***p*-value**
Gray matter	978.21	954.01	7.7E-179
White matter	671.70	680.56	3.9E-221
Cerebrospinal fluid	297.31	285.64	2.4E-101

*The TPM volumes of GM, WM and CSF obtained for the Women and Men non-parametric TPMs*.

**Table 5:** The results of the Kolmogorov-Smirnov test *p*-values of the comparisons of the Age subgroups' non-parametric TPMs.

	**Volume [ml]**		
	**Young TPMs**	**Old TPMs**	***p*-value**
Gray matter	1008.8	929.29	≈0
White matter	665.92	685.36	1.8E-192
Cerebrospinal fluid	270.91	312.07	6.6E-165

*The TPM volumes of GM, WM and CSF obtained for the Young and Old non-parametric TPMs*.

**Figure 9:**
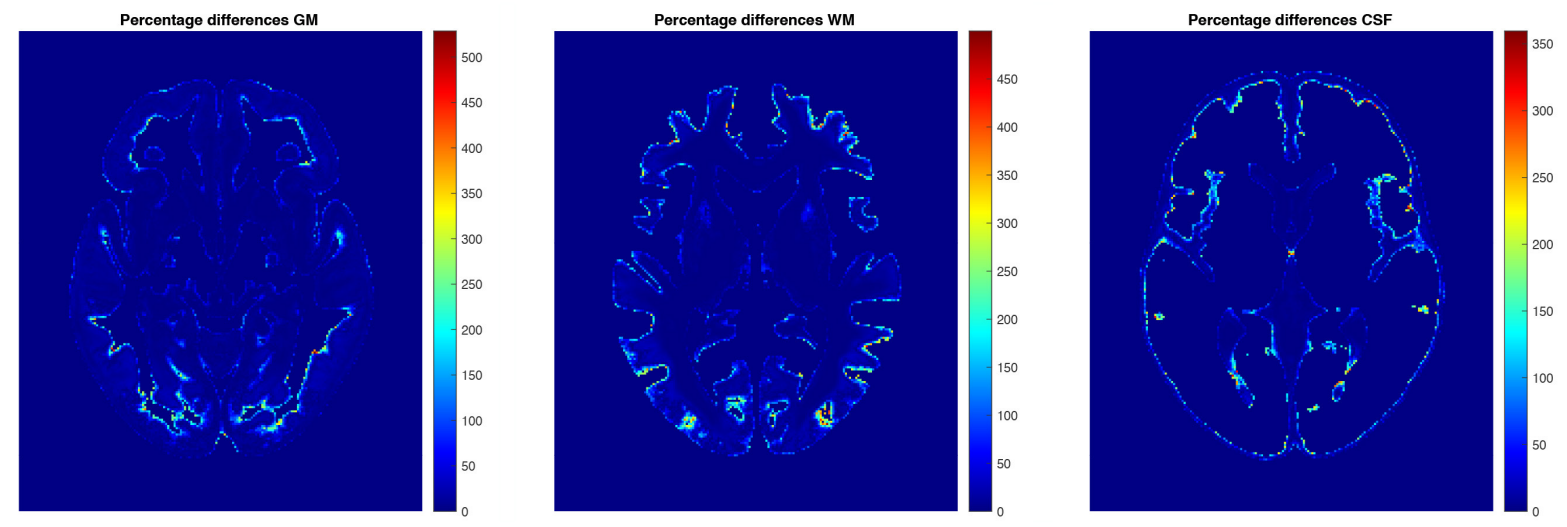
The percentage differences in GM **(left)**, WM **(middle)**, and CSF **(right)**.

The examples of axial slices for the percentage differences in the structure content between the mean and median (non-parametric 50*th* percentile) tissue proportion maps are shown in Figure 9. In the border regions of the analyzed structures, the percentage differences calculated with the application of the different statistical methods are the greatest (Figure 9). The maximum values of the percentage differences in the structure's content reach 528.5, 499.8, and 359.6%, respectively, for the GM, WM, and CSF TPMs.

### 4.3. Comparison of the Segmentation Algorithms Using Parametric and Non-parametric Tissue Probability Maps

**Figure 10:**
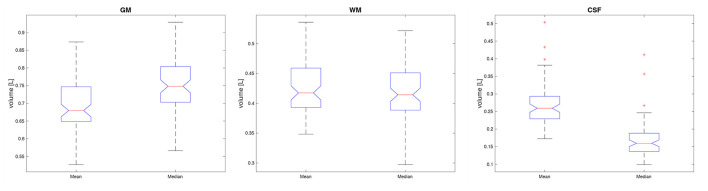
The boxplots of the volumes obtained with the parametric (mean) and non-parametric (median) TPM in the segmentation algorithm. The GM volumes are shown on the **(left)**, the WM values in the **(middle)**, and those for CSF on the **(right)** site.

In order to compare the impact of the application of the parametric or non-parametric TPM in the segmentation process, the volumetric determinants (the volumes of GM, WM, and CSF) of the brain were subjected to statistical analysis. The unified segmentation procedure was applied in Statistical Parameter Mapping (SPM) software. The volumes of GM, WM, and CSF were obtained by counting the probability in the voxels segmented using different templates. The analysis was performed in the validation cohort. The Wilcoxon signed-rank test was applied and the results are presented in Table 6 and Figure 10. As seen from the comparison, the statistically significant differences between the segmented volumes are observed for CSF and GM. The GM volumes are greater when using the non-parametric atlas in the segmentation procedure, while the CSF volumes are smaller (Table 6 and Figure 10).

**Table 6:** The differences in the volumetric values when using the parametric or non-parametric atlas in the segmentation algorithm.

	**Volume [ml]**		
	**Parametric TPM**	**Non-parametric TPM**	***p*-value**
Gray matter	679.7	748.2	2.5e-06
White matter	417.5	414.3	0.3066
Cerebrospinal fluid	259.4	159.7	4.0e-20

*The rows present the median volumes for each structure (GM/WM/CSF). The segmentation was performed with the use of the parametric and non-parametric TPMs in the validation cohort. The Wilcoxon test p-value is added in the last column*.

## 5. Discussion

The morphological features of brain ageing, to be captured effectively, require specific non-parametric brain MRI atlases (Dickie et al., 2015). The vast majority of the currently available atlases (Dickie et al., 2017) were, however, derived using parametric statistics. Our intention was to check the validity of the parametric approach in the Polish (Upper Silesian) healthy and ethnically homogeneous population. Our work provides the specific brain atlases with the associated non-parametric tissue probability maps (TPMs) for three morphological classes (GM, WM, and CSF) constructed for the sex and age sub-groups. We found that the order based (non-parametric) concept of the tissue templates is correct not only for the maps of GM in normal older subjects (Dickie et al., 2015) but for all investigated classes, regardless of age or sex.

The data distributions of the three-dimensional tissue maps (WM, GM, and CSF) were tested using the Shapiro-Wilk test. Moreover, we calculated the maps of kurtosis and skewness to check the central and outer appearance of the data distribution in the voxels. The results confirmed that in each brain structure (regardless of the studied sub-population) the data distribution is complex, skewed and leptokurtic, and apparently not Gaussian. The largest deviations from the Gaussian distribution were observed in the internal and peripheral parts of the structures. Only the regions at the border of the tissue proportion maps showed a normal population distribution.

When the assumptions for the parametric statistics are not fulfilled, choosing an inappropriate method to construct the atlas may result in masking the disease- or age-related differences in the brain structures. In consequence, this leads to a misclassification of the abnormal voxels as normal, especially in older subjects or in those with neurodegenerative diseases (Dickie et al., 2015). The largest deviations from the Gaussian distribution have been observed in the temporal and frontal regions, which are affected early in dementia (Dickie et al., 2015). In such cases, the non-parametric distributional representations of the brain are required (Dickie et al., 2015, 2017).

We created the order based atlases for the entire study sample and for the sex and age subgroups. The parametric atlases were also obtained for the sake of comparison. The non-parametric and parametric templates (their central measures) were compared using the paired sample Wilcoxon's signed-rank test to explore the influence of the chosen statistical method on the template maps. The source data with a symmetric distribution should give comparable results for the central measures (mean and median) of the content maps. However, statistically significant differences were found between the maps obtained using both measures (the maps of GM, WM, and CSF). The GM volume estimates gotten from TPM with the order-based methods were higher than when obtained with the parametric methods for all subgroups. Furthermore, the volumes of the WM and CSF TPMs calculated in the non-parametric templates were lower, regardless of the analyzed sub-population. These relationships are consistent with the skewness results from the analysis of the voxel-wise distribution, as in fact, the skewness is directly connected to it (Delucchi and Bostrom, 2004; McCluskey and Lalkhen, 2007; Limpert and Stahel, 2011; Manikandan, 2011). The regional analysis of the tissue content maps showed that the percentage differences calculated with both statistical methods are the greatest in the border regions of the analyzed structures. A strong spatial blurring of the cerebrospinal fluid in the population is visible in the form of a significantly high value of this structure content skewness (Figure 7, middle) with the long tail to the higher values of the skewness. This situation leads to an overestimation of the parametric estimators of the CSF probability maps at the expense of the volume of the gray matter structures (Delucchi and Bostrom, 2004; Limpert and Stahel, 2011; Dickie et al., 2015). Moreover, our results confirm the well known fact (Koolschijn and Crone, 2013; Kijonka et al., 2020) that the sub-population specific TPMs (Tables 4, 5) differ significantly but in fact the TMPs volumes differences are even stronger than the impact of the applied statistical measure (Table 3). The gray matter volume decreases with age, and this is accompanied by the cerebrospinal fluid volume increase (Koolschijn and Crone, 2013). In addition, a larger ratio of GM/WM in the females than in the males was observed by Koolschijn and Crone (2013). Our findings are consistent with these results as presented in the Tables 4, 5.

There are numerous examples where the description by the parametric measures is clearly misleading. This becomes obvious whenever the variable limits exist and the data that cannot exceed the specified range of the values (e.g., the probability cannot be negative or >1). Obviously, such data will be skewed, and the obtained parametric measures, compared to the order based estimates, will be shifted, thus hiding the skewed nature of the data (Delucchi and Bostrom, 2004; Limpert and Stahel, 2011). In turn, the phenomena influencing the image segmentation process are even more difficult to be described, due to the problem of the non-parametric data distribution disturbed by: the partial volume effect, random noise, and the B1 field inhomogeneities (Stanisz et al., 2005; Awate et al., 2007; Yamashiro et al., 2019). However, in this paper, we address only the non-parametric methodology in the MRI atlas creation procedure.

We also compared the impacts of applying the parametric and non-parametric TPMs on the segmentation process. The obtained volumes of GM, WM, and CSF were subjected to statistical analysis. It was performed in the validation cohort to be suitable for the objective and unbiased comparison. The Wilcoxon signed-ranks test was applied, and the results confirmed the statistically significant differences of the segmented volumes of CSF and GM obtained for different TPMs. The GM volumes are greater when using the non-parametric atlas in the segmentation procedure, while the CSF volumes are smaller. These results are consistent with the volumes of the probability maps themselves assessed before proceeding to check the impact on the classification process in the segmentation procedure.

The main limitation of the study appears to be the medium (96) sample size. However, the majority of the published atlases are mostly of modest size (the median subject number = 43) (Dickie et al., 2017). The most popular atlas, widely used in the voxel-wise analyses or support of the tissue/ROI volume segmentation—ICMB152—includes 152 healthy cases. Moreover, the available atlases are usually developed with restricted image sequences for specific processing purposes and with the underrepresented youngest and elderly populations. On the other hand, the main advantage of our study is the structure of the studied group—ethnically homogeneous, neurologically healthy, and radiologically verified. We believe that having a non-parametric group representative template, as increasing the accuracy of alignment, improving statistics, and decreasing distortions (as well as potential biases), is necessary to investigate the differences between the populations, as shown by Yang et al.—they compared two demographically matched templates, the Caucasian and Chinese standard brain atlases, and found, particularly within the language-related areas, the dramatic differences (Yang et al., 2020). It may be, thus, concluded that the combination of such factors as inherent structural variability, multi-ethnic composition, and differences in genetic influences and environmental exposures introduces a large amount of inherent variability evident in the brain morphology even in the case of the templates that are representative for age and sex (Dickie et al., 2013; Ritchie et al., 2015; Miller et al., 2016; Skorupa et al., 2017; Holla et al., 2020). Classification results obtained using parametric and non-parametric atlases do not exhaust the full prove the superiority of non-parametric methods. For example, brain age prediction is another issue worth examining. However, the atlas-based methods in brain age prediction models have not been studied until now. Especially with the use of non-parametric templates (Sajedi and Pardakhti, 2019). Therefore, the comparison of both approaches can be employed in future research to find precise brain age estimation methods with higher accuracy and thus to identify the proper statistics.

Finally, our findings for the healthy Polish (Upper Silesian) population as well as for its sex- and age-specific sub-populations suggest that the non-parametric methodology is more relevant and universal than the parametric approach. The non-parametric methods are clearly the correct choice when the assumption of normality is clearly violated, but when the parametric methods are selected, all assumptions should be satisfied. If this is not the case, it is more valid to use the non-parametric methods because they are “always valid, but not always efficient,” while the parametric ones are “always efficient, but not always valid” (Nahm, 2016). Altogether, the general conclusion is that there is no single atlas for the human brain, and there is a continuous need for age-, sex-, ethnic-, and disease-specific development of non-parametric brain representation.

## Data Availability Statement

The datasets presented in this study can be found in online repositories. The datasets (registration targets and TPMs) generated for this study can be found in the Projects section at: www.ziemowit.hpc.polsl.pl/en/ website.

## Ethics Statement

The studies involving human participants were reviewed and approved by Bioethics Committee at the Maria Sklodowska-Curie National Research Institute of Oncology Gliwice Branch. The patients/participants provided their written informed consent to participate in this study.

## Author Contributions

DB: manuscript preparation, statistical interpretation, data acquisition, optimization, and methodology selection, data processing and calculations, data segmentation, and data validation. MK: manuscript preparation, statistical analysis, statistical interpretation, optimization, and methodology selection, data processing and calculations, and data validation. KP-M: manuscript preparation, data acquisition, optimization, and methodology selection. KG: manuscript preparation and data acquisition. LZ: manuscript preparation, data validation, and radiological description of the data. MS: manuscript preparation and statistical interpretation. AS: manuscript preparation and concept discussion. All authors contributed to the article and approved the submitted version.

## Funding

Calculations were performed on the Ziemowit computer cluster in the Laboratory of Bioinformatics and Computational Biology, created in the EU Innovative Economy Programme POIG.02.01.00-00-166/08 and expanded in the POIG.02.03.01-00-040/13 project. Data analysis was partially carried out using the Biotest Platform developed within Project PBS3/B3/32/2015 financed by the Polish National Centre of Research and Development (NCBiR). This work was partially supported by the Silesian University of Technology grant no. 02/010/RGH19/0161 (DB), Silesian University of Technology statutory research funds (KP-M, AS), and Polish National Science Centre Grant no. 2020/37/B/ST6/01959 (KP-M, AS).

## Conflict of Interest

The authors declare that the research was conducted in the absence of any commercial or financial relationships that could be construed as a potential conflict of interest.

## Publisher's Note

All claims expressed in this article are solely those of the authors and do not necessarily represent those of their affiliated organizations, or those of the publisher, the editors and the reviewers. Any product that may be evaluated in this article, or claim that may be made by its manufacturer, is not guaranteed or endorsed by the publisher.
